# Correspondence

**Published:** 1875-05-15

**Authors:** 


### CORRESPONDENCE.

*London Correspondence in American Medical Weekly, April 24, 1875.*

PROFESSOR LISTER, of Edinburgh, is now publishing the results of his more recent experiences of antiseptic surgery. In spite, however, of his powerful advocacy in favor of the methodical use of antiseptic liquids in the treatment of compound fractures, etc., it is frequently demonstrated by comparative experiments that his treatment possesses little more superiority over the old and despised cold water dressing. The secret of Lister's success as a surgeon probably lies in the manipulative care and cleanliness which he practices and teaches. Next week he starts on a visit to those German and French hospitals where his method is in vogue.

A curious case of congenital skin pigmentation in a woman is reported in a foreign contemporary. On various parts of the trunk of the patient there are patches, varying in size, of a perfectly milk-white color, while the face is marked in different parts with black spots. There is no history of illness at any time, and the functions of the body are perfectly healthy and natural. The father of the woman alleges that she was born in the condition described. It must be conceded to be a very rare case, the like of which has not, as far as I am aware, been recorded in any book.

The Obstetrical Society of London will shortly commence a discussion on the relation of puerperal fever to the infective diseases and pyæmia, and especially the relation of bacteria to the pyæmic process in puerperal fever. The mortality from this fever has of late been quite excessive in this country, and if the Society will demonstrate the utility of some curative measure in its treatment, they will be doing a great service to the large body of general practitioners.

Some few years ago the administration of digitalis in typhoid fever was strongly advocated and praised, although no more cogent argument in favor of its use than that it lowered the temperature of the body, was put forward. The practice did not gain much favor when it was fairly tried, and it soon fell into desuetude. I now learn that an attempt is made to resuscitate the practice. The drug is given alone and in very large doses.

A few evenings ago a hospital surgeon described at one of the societies the cure of a pulsating tumor of the orbit (left) depending on a fracture of the base of the skull. It is some time since a case of intra-orbital aneurism has been recorded. The patient in question had a loud bruit. In the first instance compression, digital and instrumental, was perseveringly tried, but without effect. A weak solution of the perchloride of iron was then injected into the ophthalmic vein. No good result accruing, the common carotid artery was dissected out and ligatured. The man recovered quickly.

Dr. B. W. Richardson delivered a lecture last week on the causation of fever by septinous poison. He demonstrated that the secretion in the peritoneum of a patient under pyæmia was directly poisonous to the lower animals by inoculation, and that the poisonous matter could be made to combine with acids, so as to form a salt which retained the noxious qualities of the mother liquor. By a series of simple experiments he showed that the septinous poisons in the varied forms of vaccine, purulent discharge, decomposing blood, and the like, possess one and all the properties of liberating oxygen in the form of peroxide. He showed that in blood which had been rendered arterial, the oxygen is readily liberated in the presence of these disturbing causes, and that with the liberation there is a rise of temperature. In the process of liberating the oxygen, however, the liberating organic agent is itself destroyed, so that a relay of it is necessary in order to keep up the action. The theory advanced by Dr. Richardson as to the origin of fever during the presence of the septinous poisons in the body is that, absorbed in the blood, they set free an excess of oxygen until they are themselves destroyed. Then the fever remits until there is local formation of a new charge of poisonous matter and renewed absorption. The bearing of this theory on contagious fever, in all its forms, was considered with great care, and numerous clinical and therapeutical facts were cited in support of the theory, which is at present claimed to be nothing more. It is well known that Dr. Richardson has been working for years on this subject.

That bromide of potassium produces in some constitutions erythematous and acneform eruptions is tolerably well known. The eruption, as a rule, subsides on the discontinuance of the bromide. One or two cases, however, have lately been observed in which the eruption was very intractable and painful.

Araroba powder, made into an ointment, is getting into repute in the treatment of ringworm and other allied affections. It is also medicinally active, and promises to become popular as a purgative.

The great scheme of having one portal of admission into the ranks of the profession in this country has again been swamped in consequence of the disagreement of rival colleges and corporations. It has been in progress for the last five years, and after absorbing much time and attention in arranging preliminary matters, seems likely to end in smoke. Many persons pray that the government will now take the subject in hand and formulate a scheme of its own, utterly ignoring the nineteen licensing bodies that at present exist. The bitterness and animosities which the whole thing has caused are very great.

